# Effect of post-stroke cognitive impairment and dementia on stroke recurrence and functional outcomes: A systematic review and meta-analysis

**DOI:** 10.1371/journal.pone.0313633

**Published:** 2024-12-03

**Authors:** Jia Yu, Jie Wang

**Affiliations:** 1 Department of Geriatric Psychiatry, Huzhou Third Municipal Hospital, The Affiliated Hospital of Huzhou University, Huzhou City, Zhejiang Province, China; 2 Department of Internal Medicine, Huzhou Third Municipal Hospital, the Affiliated Hospital of Huzhou University, Huzhou City, Zhejiang Province, China; National center for chronic and non-communicable diesease prevention and control, CHINA

## Abstract

**Introduction:**

Post-stroke cognitive impairment (PSCI) and dementia may have a significant impact on stroke recurrence and long-term functional outcomes of patients.

**Aim:**

To investigate the potential link between PSCI and dementia, and stroke recurrence, mortality, and poor functional outcomes of stroke survivors.

**Methods:**

A systematic search across Medline, Google Scholar, and Science Direct databases was done for studies that evaluated the association of PSCI and dementia with long-term stroke outcomes. The results were expressed as pooled hazard ratios (HR) with 95% confidence intervals (CI), and heterogeneity was assessed using the I2 statistic and the Chi-square test. Subgroup analyses were performed based on the sample size, geographical location, follow-up, and type of dementia/cognitive impairment. Study quality was evaluated using the Newcastle Ottawa Scale (NOS).

**Results:**

The meta-analysis included thirteen studies. Of them, ten studies (n = 4036) reported a significant association between PSCI and stroke recurrence, with a pooled HR of 1.33 (95% CI: 1.14–1.55, I2 = 84.6%). Subgroup analysis revealed a statistically significant association between PSCI and stroke recurrence across various subrgoups. Four studies (n = 1944) demonstrated that patients with PSCI had a higher risk of poor functional outcome, with a pooled HR of 1.68 (95% CI: 1.16–2.05, I2 = 80.0%). However, the multivariate analysis did not detect a significant association between PSCI and stroke mortality, with a pooled HR of 1.50 (95% CI: 0.94–2.40, I2 = 45.9%).

**Conclusions:**

The study showed that PSCI was associated with 33% increased stroke recurrence and 68% higher rate of poor functional outcome. Our findings underscore the adverse impact of PSCI on stroke recurrence and functional outcomes, emphasizing the importance of early detection and targeted interventions to mitigate the cognitive impairment burden in stroke survivors.

## Introduction

Stroke is a leading cause of mortality and disability worldwide and an important public health issue [1]. In 2019, stroke remained the third most common cause of disability (5.7 percent of total disability-adjusted disability years [DALYs]) and the second most common cause of death worldwide (11.6% of total deaths) [2]. Recent studies have also reported that this burden has substantially increased since 1990 [2]. Stroke is also associated with a significant burden on the healthcare system due to its association with numerous long-term physical and cognitive complications in survivors [3].

Stroke is the leading cause of mortality in China, where the latest China stroke surveillance report, 2021 has stated that approximately 12.5% (95% CI 12.4–12.5%) of stroke survivors are left disabled [4, 5]. It is estimated that Post-stroke cognitive impairment (PSCI) is considered one of the main stroke complications. PSCI encompasses a spectrum of cognitive deficits ranging from mild impairment to post-stroke dementia (PSD), significantly compromising patients’ quality of life and functional independence [6]. It is defined as impairment of any of the six cognitive subgroups—visuoconstruction, attention, verbal memory, language, visual memory, and visuomotor function [7].

Studies have reported that PSCI prevalence rates range from 20% to 80%, underscoring its pervasive impact on stroke outcomes [8]. Dementia is a syndrome with a variety of underlying causes, overlapping symptoms, and diverse pathologies that impair the behavioral and cognitive capacities of patients [9]. Studies show that about 25% of stroke survivors develop PSD [10]. Moreover, estimates suggest a threefold increase in dementia incidence in patients after stroke [11]. This cognitive decline, associated with stroke, not only exacerbates functional disability but also amplifies the risk of recurrent stroke, thereby creating a vicious cycle of neurological deterioration [12].

While there is a body of research on the prevalence of PSCI and dementia in patients with stroke, studies that specifically focus on long-term follow-up of patients with PSCI/dementia and stroke are scarce. This study aims to evaluate the effect of PSCI and PSD on mortality, recurrence, and functional outcomes of stroke survivors.

## Materials and methods

A systematic search across PubMed, Science Direct, Google Scholar, and Scopus databases was done for studies published up to March 2024. Studies reporting on the effect of PSCI and PSD on selected clinical outcomes among patients with any type of stroke were reviewed by two independent authors. The review was done with the latest “Preferred reporting items for systematic reviews and meta-analyses (PRISMA)” framework [13]. The review was prospectively registered in PROSPERO (CRD42024528158). Data were extracted separately, and any conflicts of extracted information were resolved by mutual consensus. Ethical approval was not applicable since data was extracted from freely available sources. We included only articles in English in our review.

### Inclusion and exclusion criteria

- **Population:** Adult patients (>18 years of age) who presented with any type of stroke (except for Transient ischemic attacks (TIA) and intra-cerebral hemorrhages) were included as the study population. Only patients who presented with acute stroke were included, while patients who were on rehabilitative therapy were excluded. Studies that included patients diagnosed with dementia or cognitive impairment before stroke were excluded.
- **Exposure:** The exposure of interest was post-stroke cognitive impairment and dementia. We accepted any recommended definition of dementia or cognitive impairment used by the individual studies.
- **Outcome:** Our primary outcome of interest was stroke recurrence during the follow-up period. Our secondary outcomes include poor functional outcomes and all-cause mortality.
- **Study design:** We included all analytical studies (prospective, retrospective, and cross-sectional studies).

### Search strategy

We utilized medical subject heading (MeSH) terms such as: “Stroke” OR “Cerebrovascular accident” AND “Dementia” OR “Cognitive impairment” AND “Functional outcomes” OR “Mortality” OR “Recurrence” AND “Observational studies” OR “Cohort studies” OR “Prospective studies” among the included databases. The references of included studies were also reviewed to look for similar studies focusing on the same research question. The detailed search strategy is also explained in **S1 Table.**

### Data extraction and management

The two authors independently extracted data, including author details, publication year, study design, sample size, geographical location, inclusion criteria, definition of cognitive impairment/dementia, tools used to classify cognitive impairment/dementia, etc.

### Statistical analysis

We performed all statistical analyses using STATA 14.2. The collected data were double-checked for data entry errors and verified for completeness. We summarized primary (recurrence) and secondary outcomes (functional outcome and mortality) as hazards ratio (HR) with 95% confidence interval (CI). Estimates were pooled using the random effects model and Mantel-Haenszel method due to considerable clinical and high statistical heterogeneity. The effect sizes were pooled separately if univariate and multivariate estimates of HRs were provided in the individual studies. The results, in the form of pooled effect sizes, were visually depicted through forest plots. To assess publication bias, we employed funnel plots to check for the asymmetry of the funnel plot and verified it using Egger’s test [14] A p-value of less than 0.05 was considered indicative of statistical significance.

### Assessment of heterogeneity

Heterogeneity was measured using the I^2^ test, categorizing percentages into ranges: 0%–40% indicating possibly no heterogeneity, 30%–60% suggesting moderate heterogeneity, 50%–90% denoting substantial heterogeneity, and 75%–100% indicating considerable heterogeneity.

### Quality assessment of included studies

The quality of the included studies was evaluated using the Newcastle Ottawa Scale (NOS) [15]. This scale assesses study quality based on three criteria: ascertainment of outcomes, selection of study groups, and comparability. In the selection and outcome categories, a study can receive a maximum of one star for each numbered item. For comparability, a maximum of two stars can be assigned. Therefore, the NOS allows for a maximum score of nine for each study.

### Subgroup analysis

Clinical and methodological heterogeneity was further assessed using subgroup analysis wherever possible, using known explanatory variables such as the sample size, geographical location, follow-up, and type of dementia/cognitive impairment.

### Ethical statement

Ethical approval was not applicable since data was extracted from freely available sources.

## Results

### Study selection

**Fig 1:**
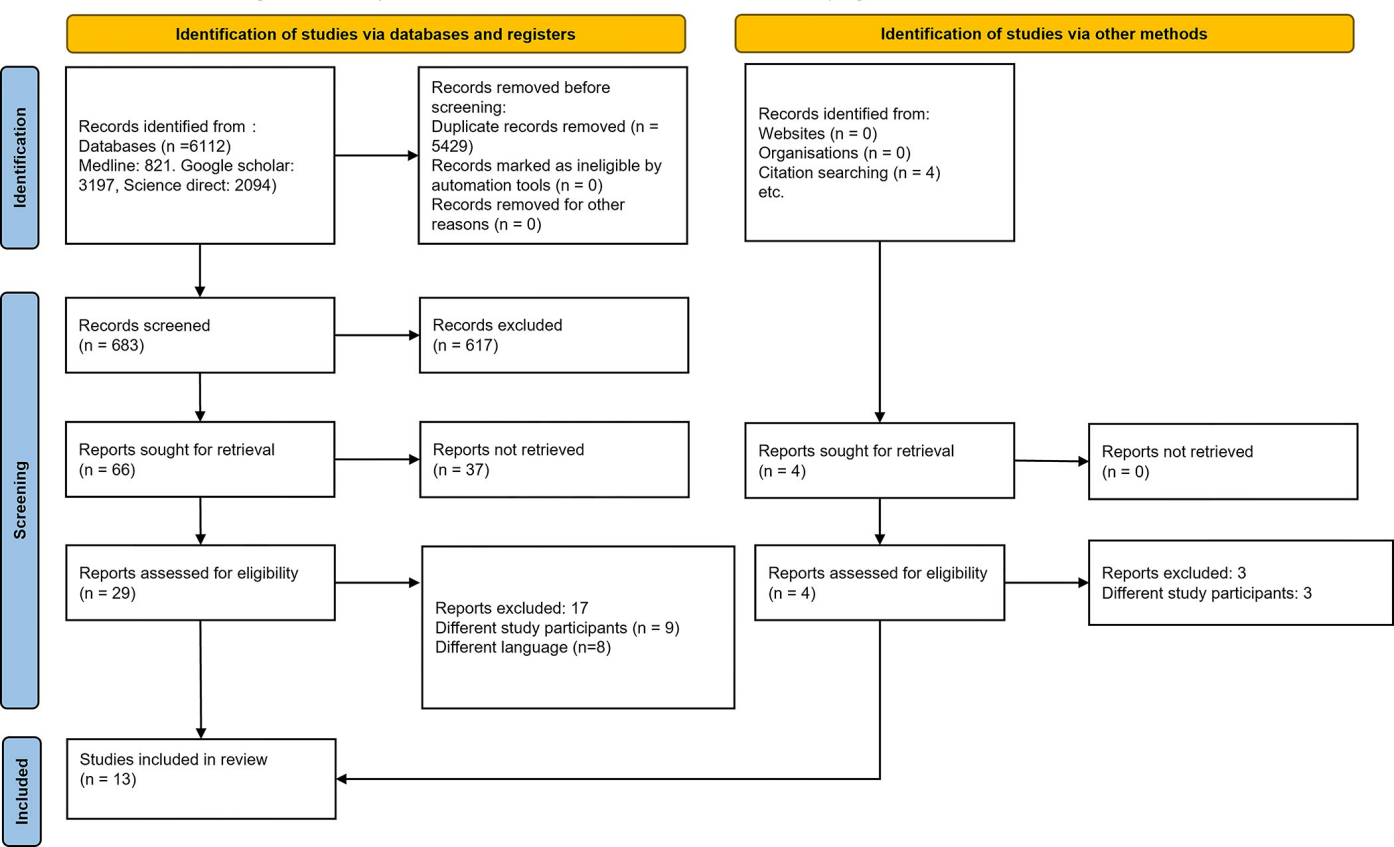
PRISMA 2020 flow diagram explaining the search flow.

The initial literature search identified a total of 6112 articles. Of them, 5429 duplicates were removed, and another 617 studies were excluded as ineligible during the secondary screening. Of the remaining 66 studies, 29 free full-text articles were retrieved. Finally, 13 articles that met eligibility criteria were included in our systematic review and meta-analysis [6, 16–27]. Ten articles reported on the effect of PSCI and/or dementia on stroke recurrence [16, 18–23, 25–27], six reported on the association of PSCI and/or dementia with mortality [6, 16, 21, 23–25], and four reported on the effect of PSCI and/or dementia on functional outcome [17, 18, 24, 27]. The PRISMA 2020 flow diagram is explained in **Fig 1.**

### Characteristics of the included studies

General characteristics of the included studies are detailed in **Table 1.** Of the 13 included studies, seven were from Asia, three were from America, and three were from Europe. The included studies had sample sizes ranging from 66 to 1528, and all 13 reported results in the English language. Four studies reported on dementia, while the nine had information on PSCI. We attempted to contact the authors individually to obtain the free full texts, and the articles were excluded if we failed to receive any response from the authors even after two consecutive mail attempts.

**Table 1:** Studies summary.

First name, year	Country	Number of participants	Study type	Tool used	Cut off	Type of PSCI/dementia	Follow up period	Age (median and range/ Mean (SD))	Inclusion and Exclusion criteria	Quality of study (NOS)
Henon 2003	France	202	Prospective	IQCODE	144	Dementia	6 months	75 (42–101)	All consecutive patients >40 years of age admitted to the Stroke	8
Li 2020	China	185	Prospective	MoCA-CS	26	Cognitive impairment	6 months	64 (54–70)	Patients aged age ≥ 18 years with acute ischemic stroke	7
Nakano 2015	Japan	66	Retrospective	HDS-R		Dementia	1 year	64 (54–70)	Patients aged age ≥ 18 years with acute ischemic stroke	7
Sibolt 2012	Finland	486	Prospective	DSM—III		Dementia	1 year	72 (66–77)	Patients aged age ≥ 18 years with acute ischemic stroke	8
Kwan 2021	USA	1528	Not provided	CASI	86	Global Impairment	3.9 ± 2.3 years	61.6 (10.7)	Adult patients with acute stroke	8
Yaghi 2020	USA	393	Not provided	MoCA-CS	26	Dementia	2 years 8 months	59.6 ± 12.6	Adult patients with acute stroke	7
Schmidt 2022	USA	246	RCT	3MS	≤ 88	Global cognitive impairment	5 years	62.96 (10.60)	Patients with ischemic stroke or TIA within 180 days of trial entry	8
Narasimhalu 2011	Singapore	419	RCT	Neuropsychological Test Battery		Cognitive impairment no dementia	3 years	54 (10)	Patients aged age ≥ 18 years with acute ischemic stroke	7
Ma 2022	China	161	Prospective	PSCI		Cognitive impairment	Not provided	65 (63, 67)	Patients with the onset form and symptoms suspicious for acute stroke and onset time within 72 hours	6
Huang 2015	China	350	Observational	TICS-m	<31	Cognitive impairment	5.8±3.2 years	42.1±7.6	Consecutive patients with a first-ever ischemic stroke	8
Liao 2022	China	1064	Cohort	MoCA	26	Cognitive impairment	1 year	58.37 ± 10.80	Patients with minor stroke	8
Dros 2023	Poland	345	Retrospective study	IQCODE	144	Dementia	5 years	78 (70–84)	Consecutive patients with acute stroke or transient ischemic attack (TIA)	7
Kwon 2019	Korea	376	RCT	MMSE	≤ 23	Cognitive impairment	4 years	71.3 (9.2)	Patients with acute stroke	6

### Excluded studies

Of the 29 full-text articles extracted, 17 were excluded during secondary screening. Nine studies were excluded because they had different study participants, and eight were reported in languages other than English.

### Effect of PSCI and/or dementia on recurrence among stroke patients

**Fig 2:**
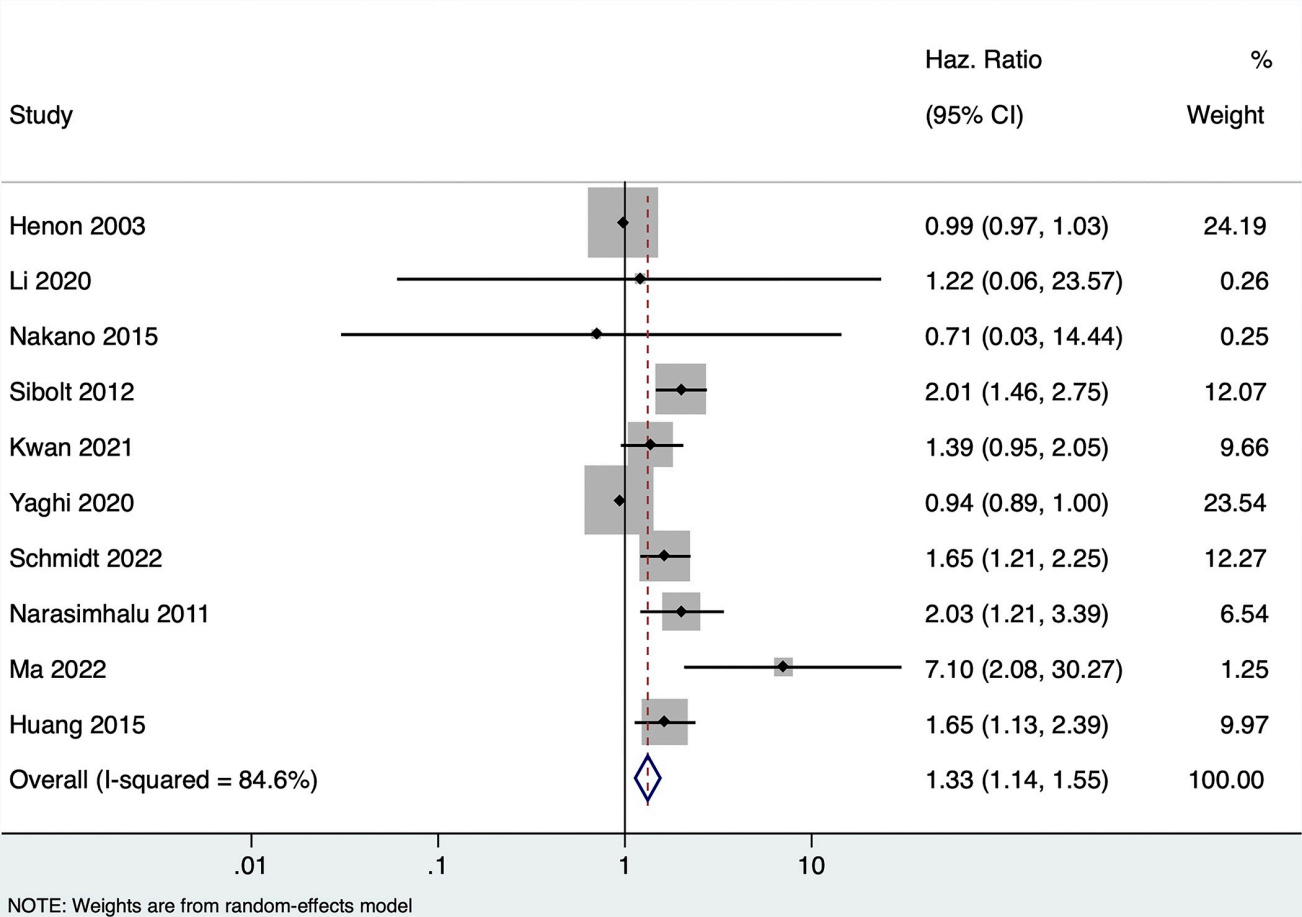
Forest plot showing the association between PSCI/dementia with recurrence (unadjusted).

Ten studies (n = 4036) reported the association (univariate) between PSCI and/or dementia and recurrence among stroke patients. Patients with any form of PSCI had a 33% higher risk of stroke recurrence during the follow-up period (pooled HR of 1.33, 95% CI: 1.14–1.55, with high heterogeneity I^2 =^ 84.6, p-value <0.001) **[Fig 2].** Only three studies reported on the multivariate-adjusted estimates, showing a non-significant association (pooled HR of 1.37, 95% CI: 0.81–2.32, with high heterogeneity I^2 =^ 87.9, p-value <0.001) **[S1 Fig]** Due to high heterogeneity, subgroup analysis was done based on the region, type of exposure, and sample size. The results showed that studies conducted in the Asian subcontinent and reporting the effect of cognitive impairment with no dementia on recurrence had a statistically significant association, with very low heterogeneity **[S2–S4 Figs]**

### Effect of PSCI and/or dementia on functional outcome among stroke patients

**Fig 3:**
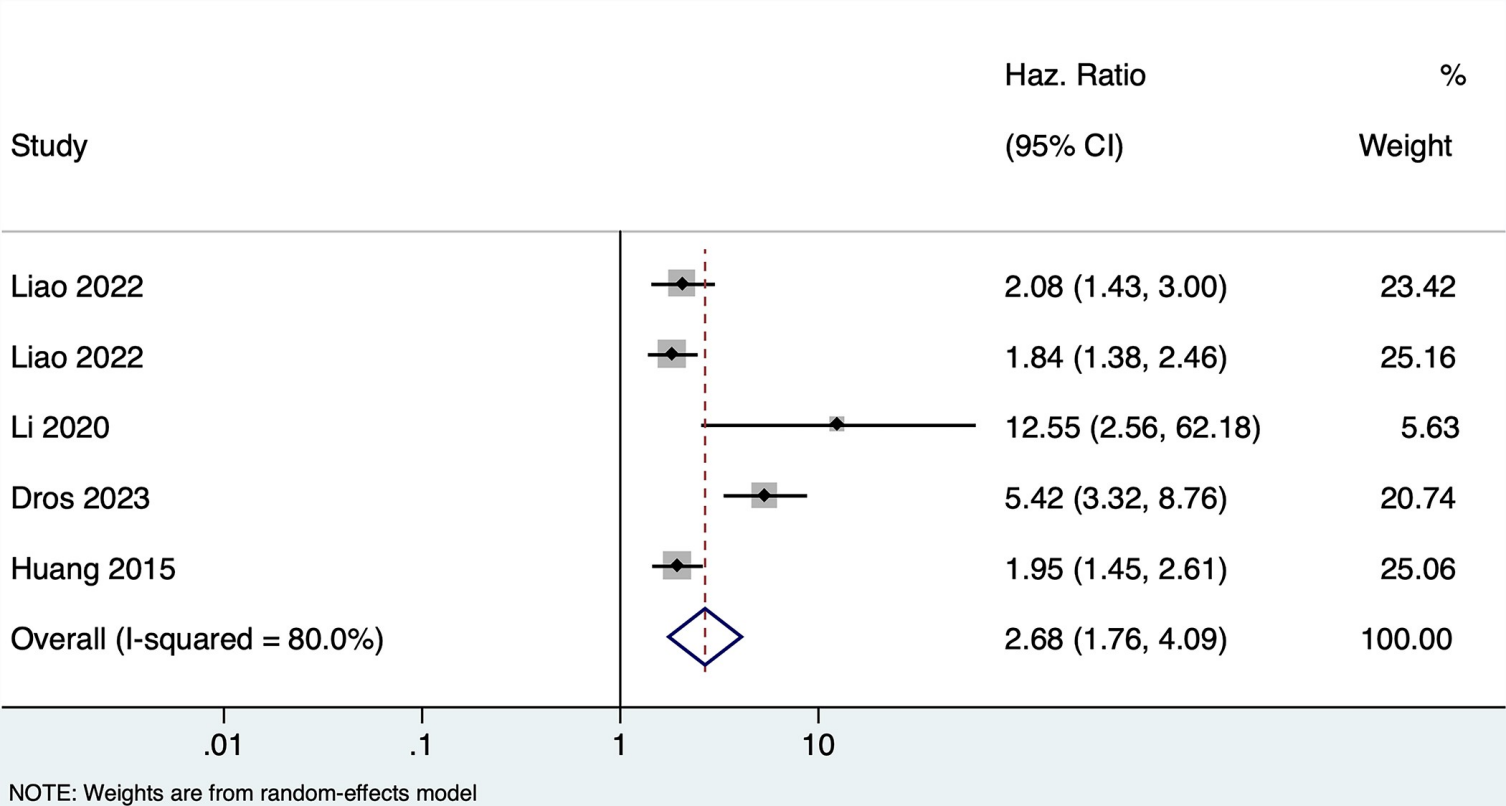
Forest plot showing the association between PSCI/dementia with functional outcome (unadjusted).

Four studies (n = 1944) reported the association between PSCI and/or dementia and functional outcomes in stroke patients. Patients with any form of PSCI had a 2.68 times higher risk of having poor functional outcomes during the follow-up period (pooled HR of 2.68, 95% CI: 1.76–4.09, with high heterogeneity I2 = 80.0, p-value <0.001) **[Fig 3].** Three studies reported on the multivariate-adjusted estimates and showed that patients with any form of PSCI had 54% higher hazards of having poor functional outcomes after adjusting for known confounders (pooled HR of 1.54, 95% CI: 1.16–2.05, with very low heterogeneity I^2 =^ 18.2, p-value 0.30) **[S5 Fig]**

### Effect of PSCI and/or dementia on mortality among stroke patients

**Fig 4:**
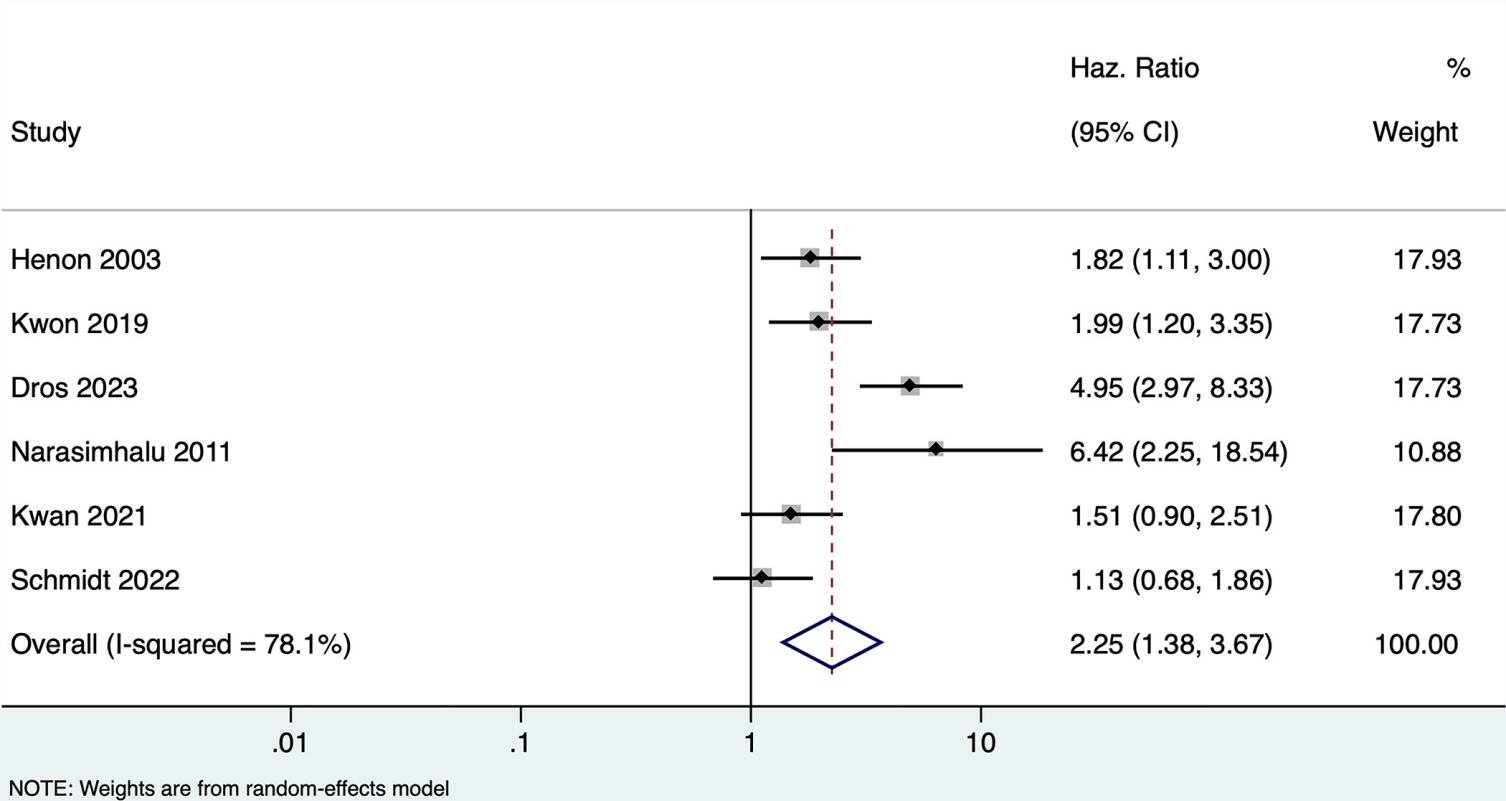
Forest plot showing the association between PSCI/dementia with mortality (unadjusted).

Six studies (n = 3323) reported the association between PSCI and/or dementia and mortality in stroke patients. Any form of PSCI was associated with 2.25 times higher risk of mortality (pooled HR of 2.25, 95% CI: 1.38–3.67, with high heterogeneity I^2 =^ 78.1, p-value <0.001). **[Fig 4]** Four studies reported on the multivariate-adjusted estimates, with a non-significant association (pooled HR of 1.50, 95% CI: 0.94–2.40, with moderate heterogeneity I^2 =^ 45.9, p value 0.13) [**S6 Fig]**

### Publication bias and risk of bias

**Fig 5:**
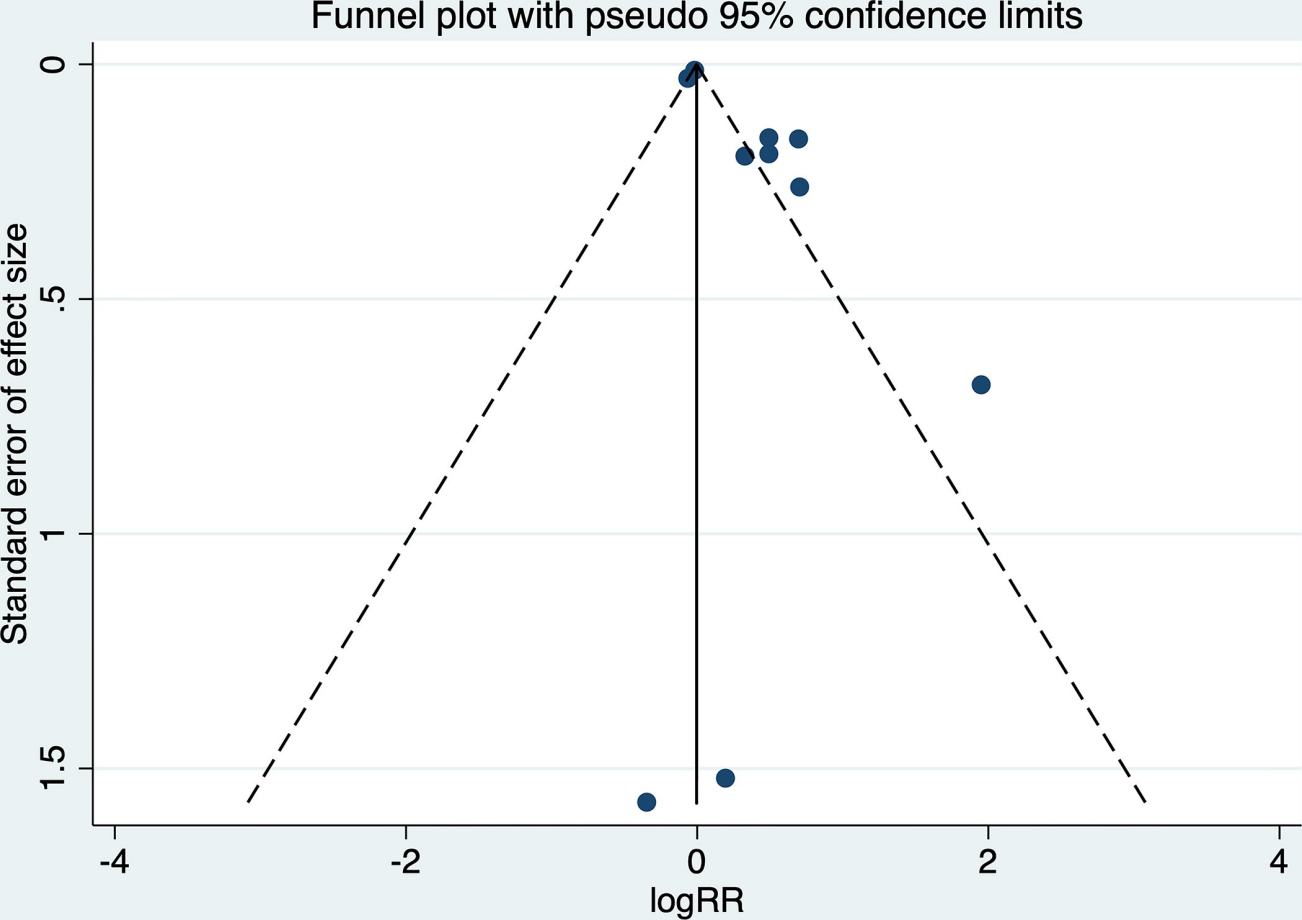
Funnel plot showing publication bias for studies showing association between PSCI/dementia with recurrence.

Presence of publication bias for association between exposure and recurrence was detected on funnel plot and Egger test (p value 0.01) [**Fig 5**]. **Table 1**and S2 Table summarizes risk of bias in the included studies, as assessed by NOS checklist for observational studies. The difference in effect estimates for various outcomes with respect to risk of bias scores is shown in **S7 Fig.**

## Discussion

Our meta-analysis included 13 studies that investigated the impact of post-stroke cognitive impairment (PSCI) and post-stroke dementia (PSD) on stroke recurrence, functional outcomes, and mortality. We observed the diagnosis of PSCI and/or dementia in stroke survivors was associated with considerably poorer functional outcomes, even after adjusting for known confounders. Our results also showed a significant association between PSCI and mortality and stroke recurrence. However, this association became insignificant in multivariable analysis. Our results provide new insights into the complex relationship between PSCI, stroke recurrence, and long-term outcomes, contributing to the growing body of research on post-stroke care.

The analysis of 10 studies (n = 4036) revealed that stroke patients with PSCI or dementia had a 33% higher risk of stroke recurrence during the follow-up period. These results were in line with the study by Hanon et al., which reported data over a 12-year follow-up [28]. In addition, a study by Cumming et al. showed that cognitive impairment is linked to poor quality of life at one-year follow-up [29]. Together, these results suggest that while cognitive impairment may indicate a higher risk of recurrence, other factors—such as vascular risk profiles, medication adherence, and the severity of the initial stroke—may contribute significantly to this risk [30, 31]. The observed high heterogeneity indicates that individual study differences, including population characteristics, study design, and methods of cognitive impairment assessment, play a role in the variability of results. Subgroup analyses provided additional insights, showing that studies conducted in the Asian subcontinent and those focused on cognitive impairment without associated dementia showed a statistically significant link to recurrence, with lower heterogeneity. This regional variation underscores the importance of considering geographic factors and sociocultural determinants in understanding the relationship between cognitive impairment and stroke outcomes [6]. Several underlying pathophysiological mechanisms can explain the observed association between PSCI and stroke recurrence. Cognitive impairment following stroke often results from cerebral vascular damage, including small vessel disease, cortical infarcts, and white matter lesions [32]. These structural alterations disrupt neural networks involved in executive function, attention, and memory, predisposing patients to impaired cerebrovascular autoregulation and increased susceptibility to recurrent ischemic events [33]. The ischemic cascade triggers excitotoxicity, oxidative stress, inflammation, and apoptosis, resulting in neuronal death and tissue damage. Following cerebral ischemia, inflammatory processes are initiated, with microglia and astrocytes releasing pro-inflammatory cytokines like IL-1β, TNF-α, and IL-6 [34, 35]. This inflammation worsens neuronal damage and contributes to cognitive decline. Furthermore, post-stroke cognitive impairment was shown to be related to white matter lesions, such as leukoaraiosis and lacunar infarcts, that may cause executive dysfunction and slower cognitive processing [36]. Additionally, cognitive impairment may compromise compensatory mechanisms such as cognitive reserve and neuroplasticity, rendering individuals more vulnerable to neurological insult and subsequent stroke recurrence [20].

Our results also showed that PSCI is strongly associated with poor functional outcomes. These results align with the existing literature suggesting that cognitive impairment following stroke not only affects cognitive domains but also interferes with physical recovery and functional independence [36, 37].

### Recommendations

The association between PSCI, dementia, and various stroke outcomes underscores the need for early detection and intervention strategies aimed at improving cognitive impairment and optimizing functional post-stroke recovery. Additionally, including cognitive outcomes in stroke trials and rehabilitation assessments could provide a more comprehensive picture of recovery. Since current stroke outcome measures, such as the Modified Rankin Scale (mRS) and Barthel Index, focus primarily on physical function and disability, they may not adequately capture the cognitive aspects of recovery. Incorporating cognitive measures, such as the Montreal Cognitive Assessment (MoCA) or Mini-Mental State Examination (MMSE), could improve the ability to predict long-term outcomes and guide individualized rehabilitation plans.

### Strengths and limitations

Our study had several strengths. To the best of our knowledge, this is the first meta-analysis that has tried to establish the relationship between PSCI/dementia and specific stroke outcomes in survivors. We have also performed additional subgroup and sensitivity analysis (using NOS), adding to the limited literature available. A large sample size of the included studies adds robustness to our results and increases their generalizability. All studies were reviewed separately by the two investigators. Nevertheless, our review has a few limitations. First, there was evidence of high heterogeneity among the included studies, which might have impacted the combined results. Although subgroup analysis and sensitivity analyses were used to adjust for heterogeneity, the underlying variability in methodologies, tools used for defining cognitive impairment and dementia, and duration of follow-up should be considered when interpreting the results. Additionally, we need to acknowledge the potential impact of the publication bias on the interpretation of our results. Lastly, this review included only free full-text English-language articles, while the grey literature was excluded.

### Conclusions

In summary, our systematic review and meta-analysis highlight the adverse effect of post-stroke cognitive impairment and dementia on stroke recurrence and functional outcomes. Healthcare providers should prioritize routine cognitive screening in stroke survivors, utilizing validated tools to detect early signs of cognitive impairment. Early identification of cognitive impairment will enable prompt initiation of targeted interventions, including cognitive rehabilitation, pharmacological management, patient education, and lifestyle modifications. Our study also emphasizes the need for a multidisciplinary approach to reduce the risk of adverse stroke outcomes. Future research must also focus on standardized assessment scales and adopt newer interventions to improve the cognitive function of stroke survivors.

## Supporting information

S1 Fig Forest plot showing the association between PSCI/dementia with recurrence (adjusted). (TIF)

S2 Fig Forest plot showing the association between PSCI/dementia with recurrence grouped by continent. (TIF)

S3 Fig Forest plot showing the association between PSCI/dementia with recurrence grouped by type. (TIF)

S4 Fig Forest plot showing the association between PSCI/dementia with recurrence grouped by sample size. (TIF)

S5 Fig Forest plot showing the association between PSCI/dementia with functional outcome (adjusted). (TIF)

S6 Fig Forest plot showing the association between PSCI/dementia with mortality (adjusted). (TIF)

S7 Fig Forest plot showing the association between PSCI/dementia with recurrence grouped by NOS. (TIF)

S1 Table Search strategy. (DOCX)

S2 Table Components of Newcastle-Ottawa Scale across the included studies (NOS). (DOCX)

S1 File PRISMA checklist. (DOCX)

S2 File A numbered table of all studies identified in the literature search. (DOCX)

S3 File All data extracted from the primary research sources for the systematic review and/or meta-analysis. (DOCX)

S4 File The completed risk of bias and quality/certainty assessments for each study or outcome. (DOCX)
