# Supplementary figures and images for: Effect of post-stroke cognitive impairment and dementia on stroke recurrence and functional outcomes: A systematic review and meta-analysis
Source: PLoS One. 2024 Dec 3;19(12):e0313633. doi: 10.1371/journal.pone.0313633 (PMC11614207; doi:10.1371/journal.pone.0313633)

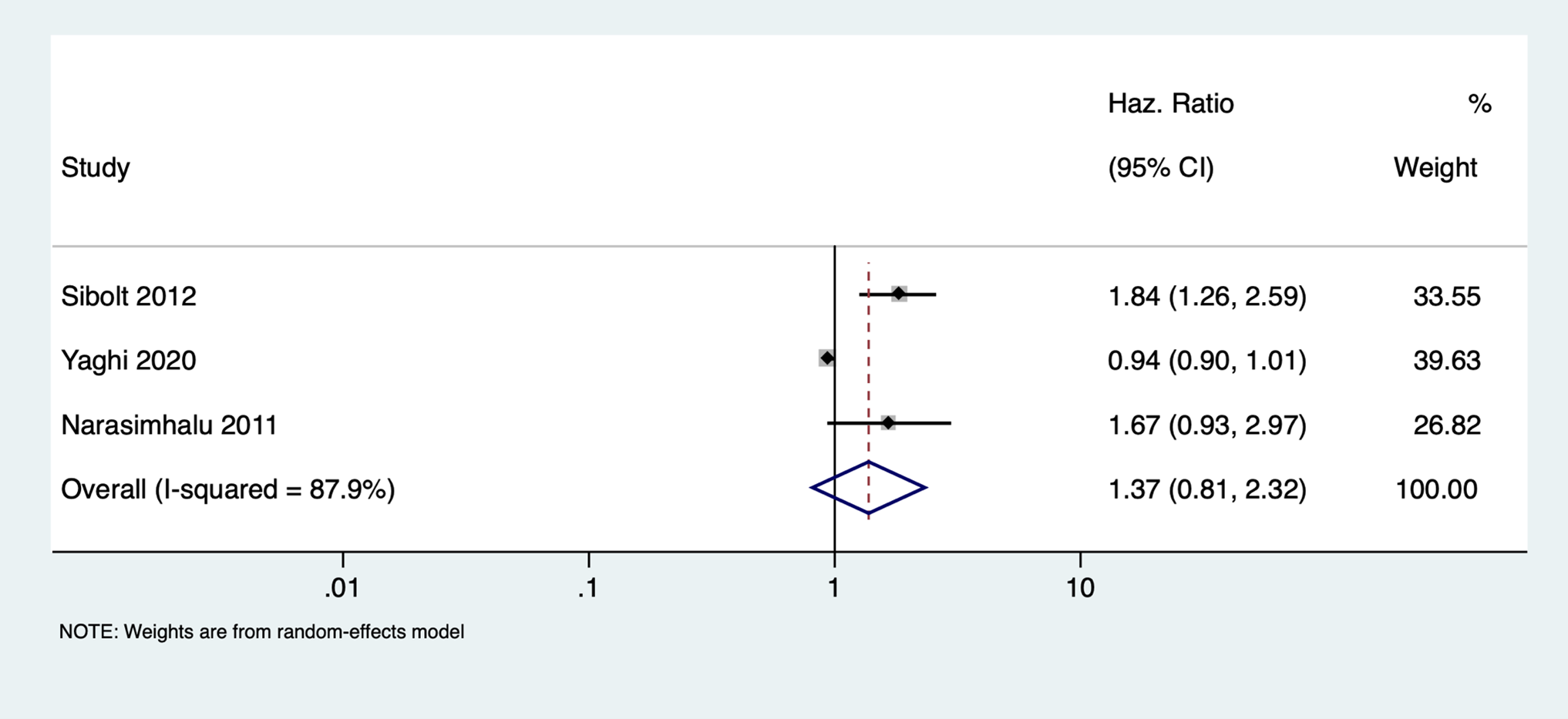

Supplement: S1 Fig — (TIF) [file pone.0313633.s001.tif]

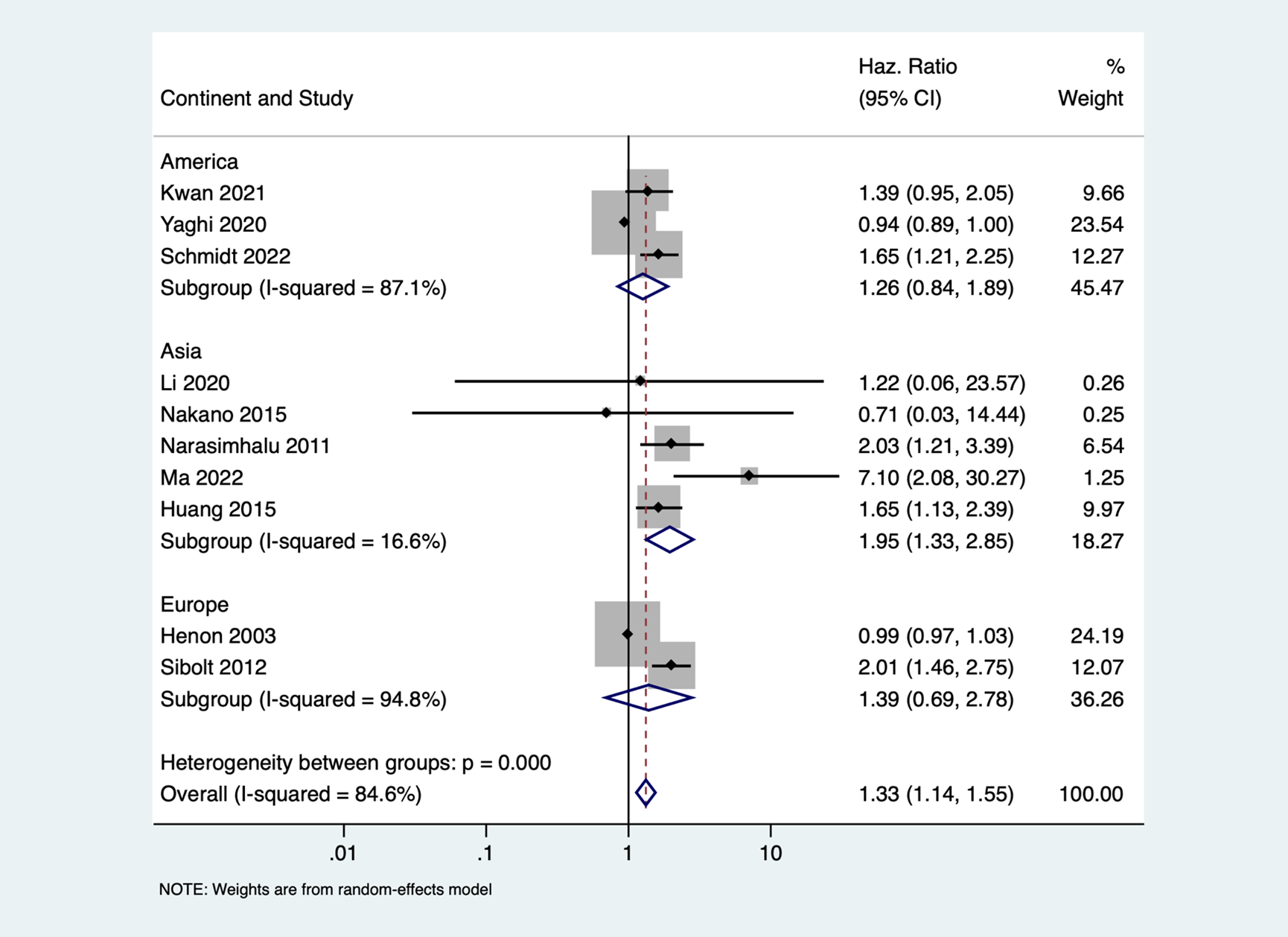

Supplement: S2 Fig — (TIF) [file pone.0313633.s002.tif]

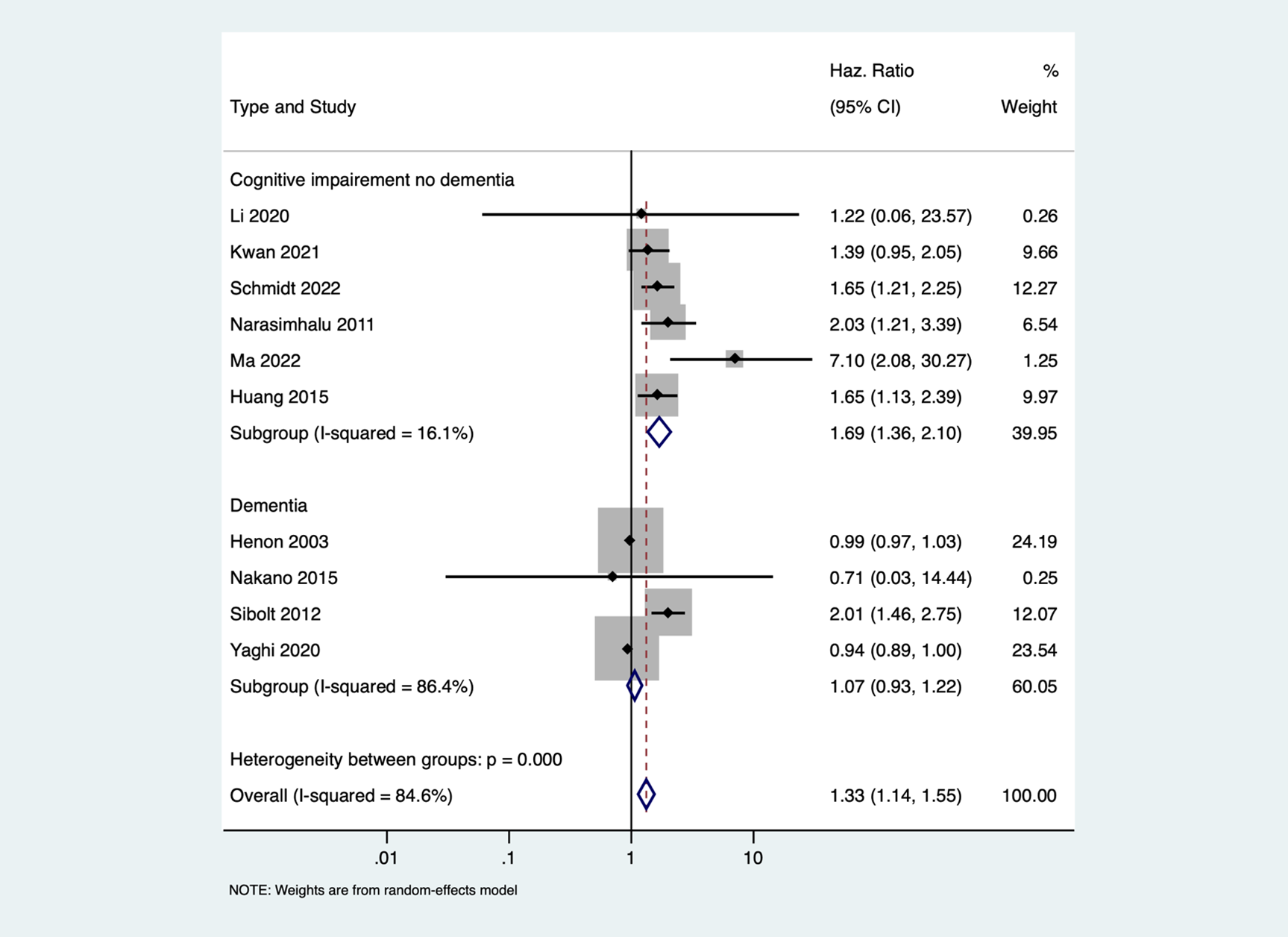

Supplement: S3 Fig — (TIF) [file pone.0313633.s003.tif]

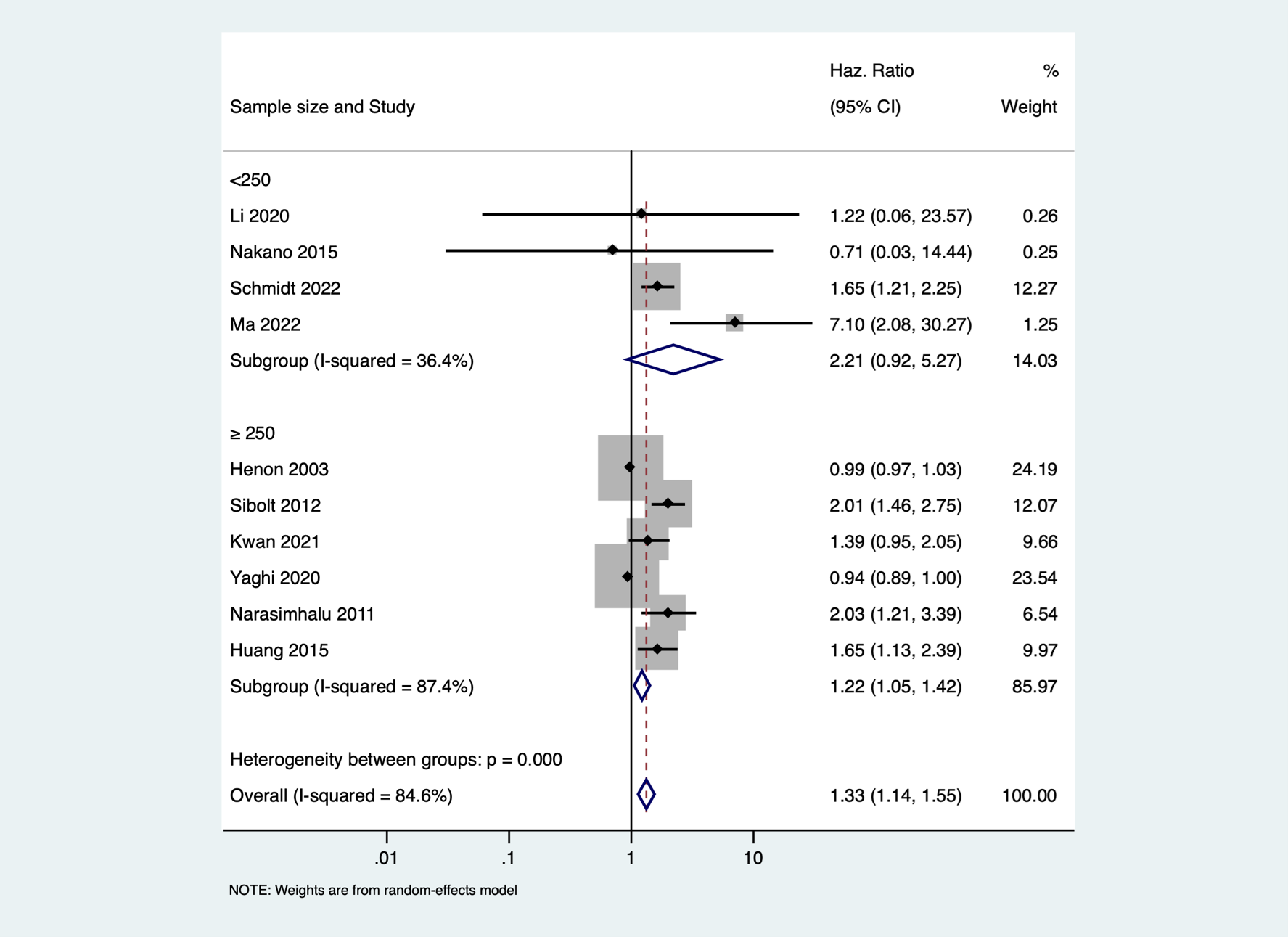

Supplement: S4 Fig — (TIF) [file pone.0313633.s004.tif]

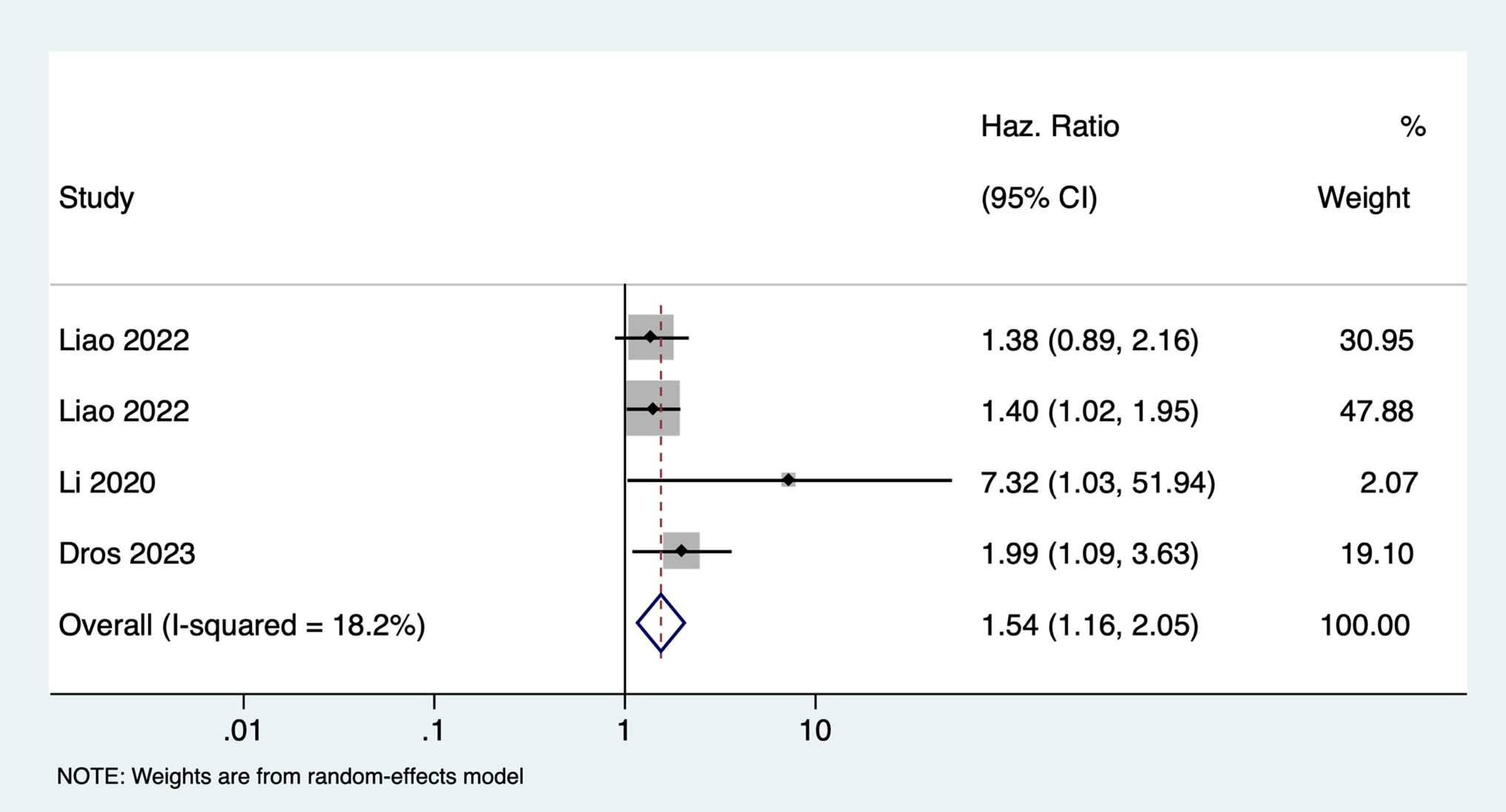

Supplement: S5 Fig — (TIF) [file pone.0313633.s005.tif]

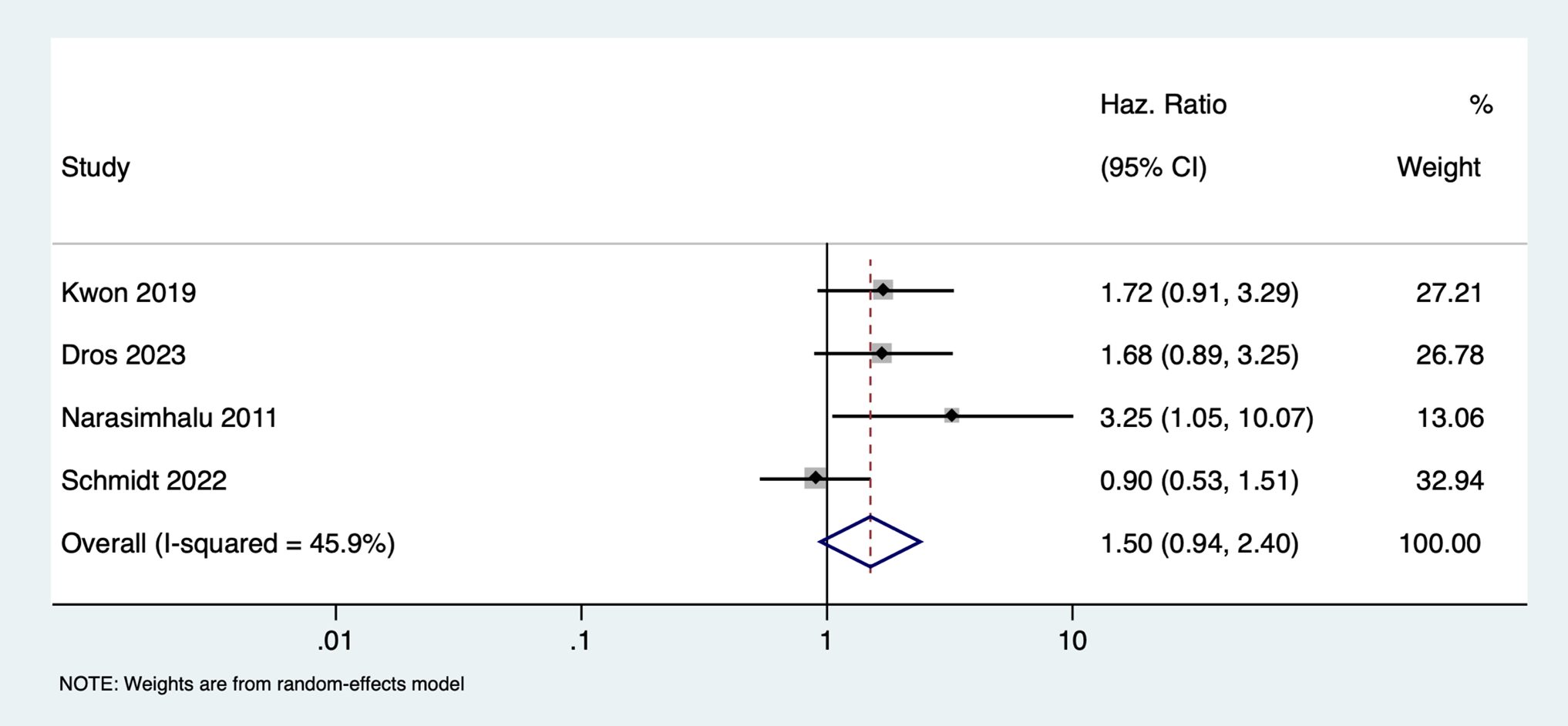

Supplement: S6 Fig — (TIF) [file pone.0313633.s006.tif]

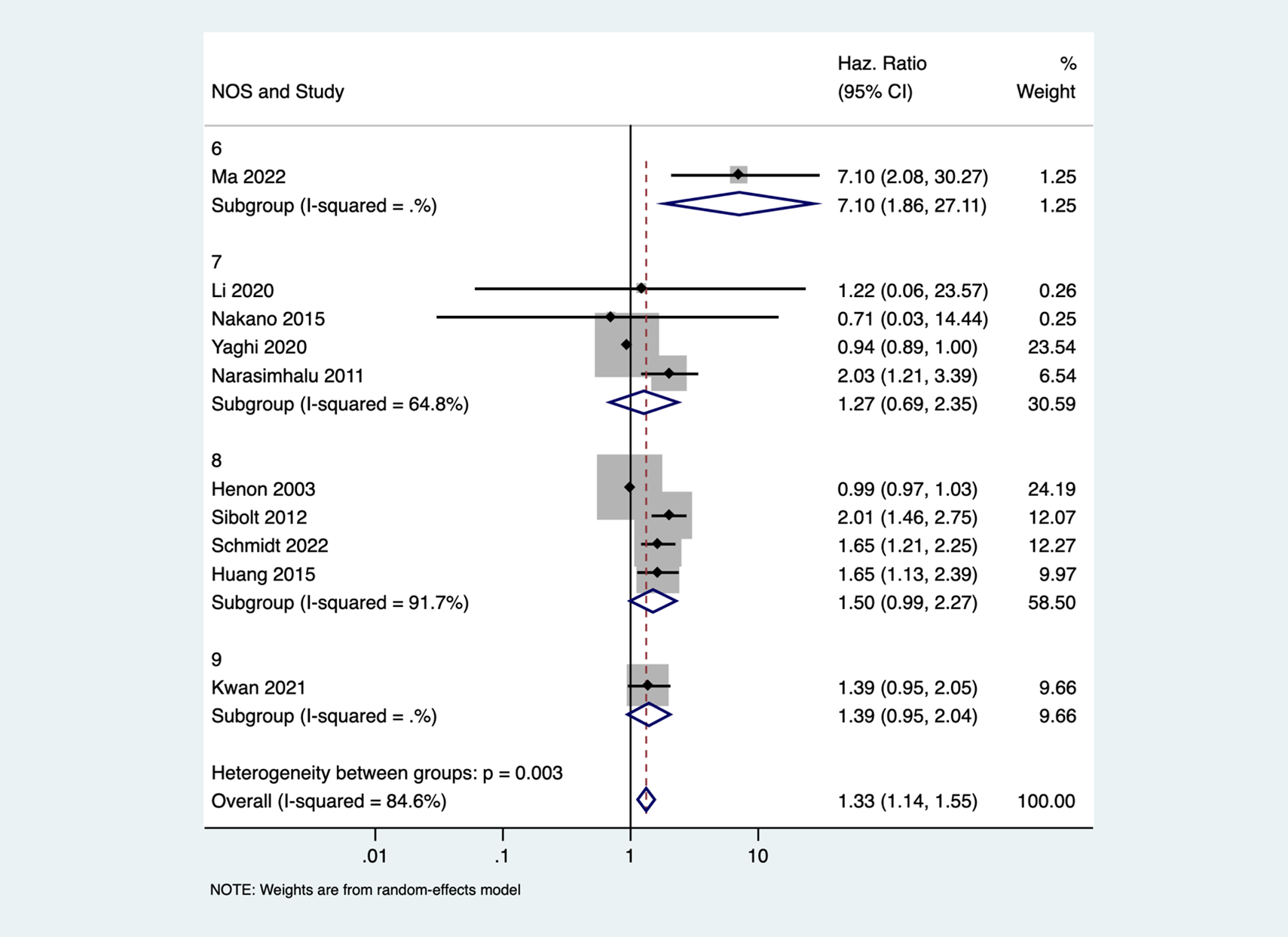

Supplement: S7 Fig — (TIF) [file pone.0313633.s007.tif]
