# Supplementary material for: Effect of post-stroke cognitive impairment and dementia on stroke recurrence and functional outcomes: A systematic review and meta-analysis
Source: PLoS One. 2024 Dec 3;19(12):e0313633. doi: 10.1371/journal.pone.0313633 (PMC11614207; doi:10.1371/journal.pone.0313633)
Supplement: S1 Table — (DOCX) [file pone.0313633.s008.docx]

**S1 Table: Search Strategy**

| No | Keywords | Details |
| --- | --- | --- |
| 1 | **(post-stroke cognitive impairment) AND (stroke recurrence)** | "post-stroke"[All Fields] AND ("cognitive dysfunction"[MeSH Terms] OR ("cognitive"[All Fields] AND "dysfunction"[All Fields]) OR "cognitive dysfunction"[All Fields] OR ("cognitive"[All Fields] AND "impairment"[All Fields]) OR "cognitive impairment"[All Fields]) AND (("stroke"[MeSH Terms] OR "stroke"[All Fields] OR "strokes"[All Fields] OR "stroke s"[All Fields]) AND ("recurrance"[All Fields] OR "recurrence"[MeSH Terms] OR "recurrence"[All Fields] OR "recurrences"[All Fields] OR "recurrencies"[All Fields] OR "recurrency"[All Fields] OR "recurrent"[All Fields] OR "recurrently"[All Fields] OR "recurrents"[All Fields])) |
| 2 | **((post-stroke cognitive impairment) AND (stroke recurrence)) AND (Dementia)** | "post-stroke"[All Fields] AND ("cognitive dysfunction"[MeSH Terms] OR ("cognitive"[All Fields] AND "dysfunction"[All Fields]) OR "cognitive dysfunction"[All Fields] OR ("cognitive"[All Fields] AND "impairment"[All Fields]) OR "cognitive impairment"[All Fields]) AND (("stroke"[MeSH Terms] OR "stroke"[All Fields] OR "strokes"[All Fields] OR "stroke s"[All Fields]) AND ("recurrance"[All Fields] OR "recurrence"[MeSH Terms] OR "recurrence"[All Fields] OR "recurrences"[All Fields] OR "recurrencies"[All Fields] OR "recurrency"[All Fields] OR "recurrent"[All Fields] OR "recurrently"[All Fields] OR "recurrents"[All Fields])) AND ("dementia"[MeSH Terms] OR "dementia"[All Fields] OR "dementias"[All Fields] OR "dementia s"[All Fields]) |
| 3 | **((frailty) AND (stroke)) AND ((poor functional outcome) OR (Mortality))** | ("frailty"[MeSH Terms] OR "frailty"[All Fields] OR "frailties"[All Fields]) AND ("stroke"[MeSH Terms] OR "stroke"[All Fields] OR "strokes"[All Fields] OR "stroke s"[All Fields]) AND ((("poverty"[MeSH Terms] OR "poverty"[All Fields] OR "poor"[All Fields]) AND ("functional"[All Fields] OR "functional s"[All Fields] OR "functionalities"[All Fields] OR "functionality"[All Fields] OR "functionalization"[All Fields] OR "functionalizations"[All Fields] OR "functionalize"[All Fields] OR "functionalized"[All Fields] OR "functionalizes"[All Fields] OR "functionalizing"[All Fields] OR "functionally"[All Fields] OR "functionals"[All Fields] OR "functioned"[All Fields] OR "functioning"[All Fields] OR "functionings"[All Fields] OR "functions"[All Fields] OR "physiology"[MeSH Subheading] OR "physiology"[All Fields] OR "function"[All Fields] OR "physiology"[MeSH Terms]) AND ("outcome"[All Fields] OR "outcomes"[All Fields])) OR ("mortality"[MeSH Terms] OR "mortality"[All Fields] OR "mortalities"[All Fields] OR "mortality"[MeSH Subheading])) |
| 4 | **((post-stroke cognitive impairment) OR (Dementia)) AND (stroke recurrence)** | (("post-stroke"[All Fields] AND ("cognitive dysfunction"[MeSH Terms] OR ("cognitive"[All Fields] AND "dysfunction"[All Fields]) OR "cognitive dysfunction"[All Fields] OR ("cognitive"[All Fields] AND "impairment"[All Fields]) OR "cognitive impairment"[All Fields])) OR ("dementia"[MeSH Terms] OR "dementia"[All Fields] OR "dementias"[All Fields] OR "dementia s"[All Fields])) AND (("stroke"[MeSH Terms] OR "stroke"[All Fields] OR "strokes"[All Fields] OR "stroke s"[All Fields]) AND ("recurrance"[All Fields] OR "recurrence"[MeSH Terms] OR "recurrence"[All Fields] OR "recurrences"[All Fields] OR "recurrencies"[All Fields] OR "recurrency"[All Fields] OR "recurrent"[All Fields] OR "recurrently"[All Fields] OR "recurrents"[All Fields])) |
| 5 | **Cognitive Dysfunction"[MeSH] OR cognitive impairment[tiab] OR cognitive decline[tiab] OR cognition disorders[tiab] OR dementia[MeSH] OR dementia[tiab]** | "cognitive dysfunction"[MeSH Terms] OR "cognitive impairment"[Title/Abstract] OR "cognitive decline"[Title/Abstract] OR "cognition disorders"[Title/Abstract] OR "dementia"[MeSH Terms] OR "dementia"[Title/Abstract] |
| 6 | **Recurrence [MeSH] OR recurrence[tiab] OR repeated stroke[tiab] OR stroke recurrence[tiab] OR second stroke[tiab]** | "recurrence"[MeSH Terms] OR "recurrence"[Title/Abstract] OR "repeated stroke"[Title/Abstract] OR "stroke recurrence"[Title/Abstract] OR "second stroke"[Title/Abstract] |
| 7 | **Functional Outcome [MeSH] OR functional outcomes[tiab] OR functional recovery[tiab] OR activities of daily living[MeSH] OR ADL[tiab] OR disability[tiab] OR functional status[tiab]** | "functional outcomes"[Title/Abstract] OR "functional recovery"[Title/Abstract] OR "activities of daily living"[MeSH Terms] OR "ADL"[Title/Abstract] OR "disability"[Title/Abstract] OR "functional status"[Title/Abstract] |
| 5 | **#1 AND #2 AND #3 OR (#4 OR #5 OR #6) AND #4** | "post-stroke"[All Fields] AND ("cognitive dysfunction"[MeSH Terms] OR ("cognitive"[All Fields] AND "dysfunction"[All Fields]) OR "cognitive dysfunction"[All Fields] OR ("cognitive"[All Fields] AND "impairment"[All Fields]) OR "cognitive impairment"[All Fields]) AND (("stroke"[MeSH Terms] OR "stroke"[All Fields] OR "strokes"[All Fields] OR "stroke s"[All Fields]) AND ("recurrance"[All Fields] OR "recurrence"[MeSH Terms] OR "recurrence"[All Fields] OR "recurrences"[All Fields] OR "recurrencies"[All Fields] OR "recurrency"[All Fields] OR "recurrent"[All Fields] OR "recurrently"[All Fields] OR "recurrents"[All Fields])) AND ("post-stroke"[All Fields] AND ("cognitive dysfunction"[MeSH Terms] OR ("cognitive"[All Fields] AND "dysfunction"[All Fields]) OR "cognitive dysfunction"[All Fields] OR ("cognitive"[All Fields] AND "impairment"[All Fields]) OR "cognitive impairment"[All Fields]) AND (("stroke"[MeSH Terms] OR "stroke"[All Fields] OR "strokes"[All Fields] OR "stroke s"[All Fields]) AND ("recurrance"[All Fields] OR "recurrence"[MeSH Terms] OR "recurrence"[All Fields] OR "recurrences"[All Fields] OR "recurrencies"[All Fields] OR "recurrency"[All Fields] OR "recurrent"[All Fields] OR "recurrently"[All Fields] OR "recurrents"[All Fields])) AND ("dementia"[MeSH Terms] OR "dementia"[All Fields] OR "dementias"[All Fields] OR "dementia s"[All Fields])) AND (("post-stroke"[All Fields] AND ("cognitive dysfunction"[MeSH Terms] OR ("cognitive"[All Fields] AND "dysfunction"[All Fields]) OR "cognitive dysfunction"[All Fields] OR ("cognitive"[All Fields] AND "impairment"[All Fields]) OR "cognitive impairment"[All Fields]) AND (("stroke"[MeSH Terms] OR "stroke"[All Fields] OR "strokes"[All Fields] OR "stroke s"[All Fields]) AND ("recurrance"[All Fields] OR "recurrence"[MeSH Terms] OR "recurrence"[All Fields] OR "recurrences"[All Fields] OR "recurrencies"[All Fields] OR "recurrency"[All Fields] OR "recurrent"[All Fields] OR "recurrently"[All Fields] OR "recurrents"[All Fields]))) OR ("post-stroke"[All Fields] AND ("cognitive dysfunction"[MeSH Terms] OR ("cognitive"[All Fields] AND "dysfunction"[All Fields]) OR "cognitive dysfunction"[All Fields] OR ("cognitive"[All Fields] AND "impairment"[All Fields]) OR "cognitive impairment"[All Fields]) AND (("stroke"[MeSH Terms] OR "stroke"[All Fields] OR "strokes"[All Fields] OR "stroke s"[All Fields]) AND ("recurrance"[All Fields] OR "recurrence"[MeSH Terms] OR "recurrence"[All Fields] OR "recurrences"[All Fields] OR "recurrencies"[All Fields] OR "recurrency"[All Fields] OR "recurrent"[All Fields] OR "recurrently"[All Fields] OR "recurrents"[All Fields])) AND ("dementia"[MeSH Terms] OR "dementia"[All Fields] OR "dementias"[All Fields] OR "dementia s"[All Fields]))) AND ((("post-stroke"[All Fields] AND ("cognitive dysfunction"[MeSH Terms] OR ("cognitive"[All Fields] AND "dysfunction"[All Fields]) OR "cognitive dysfunction"[All Fields] OR ("cognitive"[All Fields] AND "impairment"[All Fields]) OR "cognitive impairment"[All Fields])) OR ("dementia"[MeSH Terms] OR "dementia"[All Fields] OR "dementias"[All Fields] OR "dementia s"[All Fields])) AND (("stroke"[MeSH Terms] OR "stroke"[All Fields] OR "strokes"[All Fields] OR "stroke s"[All Fields]) AND ("recurrance"[All Fields] OR "recurrence"[MeSH Terms] OR "recurrence"[All Fields] OR "recurrences"[All Fields] OR "recurrencies"[All Fields] OR "recurrency"[All Fields] OR "recurrent"[All Fields] OR "recurrently"[All Fields] OR "recurrents"[All Fields]))) |

Information source: Pubmed, Google Scholar, Science Direct
