# Supplementary material for: Effect of post-stroke cognitive impairment and dementia on stroke recurrence and functional outcomes: A systematic review and meta-analysis
Source: PLoS One. 2024 Dec 3;19(12):e0313633. doi: 10.1371/journal.pone.0313633 (PMC11614207; doi:10.1371/journal.pone.0313633)
Supplement: S2 Table — (DOCX) [file pone.0313633.s009.docx]

**S2 Table: Components of Newcastle-Ottawa Scale across the included studies (NOS)**

| Study | Representativeness of the exposed cohort (1) | Selection of the non-exposed cohort (1) | Ascertainment of exposure (1) | Demonstration that outcome of interest was not present at start of study (1) | Comparability of cohorts on the basis of the design or analysis (2) | Assessment of outcome (1) | Was follow up long enough for outcomes to occur (1) | Adequacy of follow up of cohorts (1) | Total |
| --- | --- | --- | --- | --- | --- | --- | --- | --- | --- |
| Henon 2003 | 1 | 1 | 1 | 1 | 2 | 1 | 0 | 1 | 8 |
| Li 2020 | 1 | 1 | 1 | 1 | 2 | 1 | 0 | 0 | 7 |
| Nakano 2015 | 1 | 1 | 1 | 1 | 0 | 1 | 1 | 1 | 7 |
| Sibolt 2012 | 1 | 1 | 1 | 1 | 2 | 1 | 1 | 1 | 8 |
| Kwan 2021 | 0 | 1 | 1 | 1 | 2 | 1 | 1 | 1 | 8 |
| Yaghi 2020 | 1 | 0 | 1 | 1 | 2 | 1 | 1 | 0 | 7 |
| Schmidt 2022 | 1 | 1 | 0 | 1 | 2 | 1 | 1 | 1 | 8 |
| Narasimhalu 2011 | 1 | 1 | 1 | 1 | 0 | 1 | 1 | 1 | 7 |
| Ma 2022 | 1 | 1 | 1 | 1 | 0 | 1 | 0 | 1 | 6 |
| Huang 2015 | 1 | 1 | 1 | 0 | 2 | 1 | 1 | 1 | 8 |
| Liao 2022 | 1 | 1 | 1 | 1 | 2 | 1 | 0 | 1 | 8 |
| Dros 2023 | 1 | 1 | 1 | 1 | 0 | 1 | 1 | 1 | 7 |
| Kwon 2019 | 1 | 1 | 1 | 0 | 0 | 1 | 1 | 1 | 6 |
