# Supplementary material for: Effect of post-stroke cognitive impairment and dementia on stroke recurrence and functional outcomes: A systematic review and meta-analysis
Source: PLoS One. 2024 Dec 3;19(12):e0313633. doi: 10.1371/journal.pone.0313633 (PMC11614207; doi:10.1371/journal.pone.0313633)
Supplement: S2 File — (DOCX) [file pone.0313633.s011.docx]

A numbered table of all studies identified in the literature search, including those that were excluded from the analyses.
a. For every excluded study, the table should list the reason(s) for exclusion.
b. If any of the included studies are unpublished, include a link (URL) to the primary source or detailed information about how the content can be accessed.

| Study/Citation | Included/Excluded | Reasons for exclusion |
| --- | --- | --- |
| Kwon HS, Lee D, Lee MH, Yu S, Lim J-S, Yu K-H, et al. Post-stroke cognitive impairment as an independent predictor of ischemic stroke recurrence: PICASSO sub-study. J Neurol. 2020;267: 688–693. doi:10.1007/s00415-019-09630-4 | Included |  |
| Hénon H, Vroylandt P, Durieu I, Pasquier F, Leys D. Leukoaraiosis more than dementia is a predictor of stroke recurrence. Stroke. 2003;34: 2935–2940. doi:10.1161/01.STR.0000103747.58719.59 | Included |  |
| Liao X, Zuo L, Dong Y, Pan Y, Yan H, Meng X, et al. Persisting cognitive impairment predicts functional dependence at 1 year after stroke and transient ischemic attack: a longitudinal, cohort study. BMC Geriatr. 2022;22: 1009. doi:10.1186/s12877-022-03609-z | Included |  |
| Li J, Wang J, Wu B, Xu H, Wu X, Zhou L, et al. Association Between Early Cognitive Impairment and Midterm Functional Outcomes Among Chinese Acute Ischemic Stroke Patients: A Longitudinal Study. Front Neurol. 2020;11: 20. doi:10.3389/fneur.2020.00020 | Included |  |
| Nakano Y, Deguchi K, Yamashita T, Morihara R, Matsuzono K, Kawahara Y, et al. High Incidence of Dementia Conversion than Stroke Recurrence in Poststroke Patients of Late Elder Society. J Stroke Cerebrovasc Dis. 2015;24: 1621–1628. doi:10.1016/j.jstrokecerebrovasdis.2015.03.037 | Included |  |
| Sibolt G, Curtze S, Melkas S, Putaala J, Pohjasvaara T, Kaste M, et al. Poststroke dementia is associated with recurrent ischaemic stroke. J Neurol Neurosurg Psychiatry. 2013;84: 722–726. doi:10.1136/jnnp-2012-304084 | Included |  |
| Kwan A, Wei J, Dowling NM, Power MC, Nadareishvili Z, SPS3 Study Group. Cognitive Impairment after Lacunar Stroke and the Risk of Recurrent Stroke and Death. Cerebrovasc Dis. 2021;50: 383–389. doi:10.1159/000514261 | Included |  |
| Yaghi S, Cotsonis G, de Havenon A, Prahbakaran S, Romano JG, Lazar RM, et al. Poststroke Montreal Cognitive Assessment and Recurrent Stroke in Patients With Symptomatic Intracranial Atherosclerosis. J Stroke Cerebrovasc Dis. 2020;29: 104663. doi:10.1016/j.jstrokecerebrovasdis.2020.104663 | Included |  |
| Schmidt K, Power MC, Ciarleglio A, Nadareishvili Z, IRIS Study Group. Post-stroke cognitive impairment and the risk of stroke recurrence and death in patients with insulin resistance. J Stroke Cerebrovasc Dis. 2022;31: 106744. doi:10.1016/j.jstrokecerebrovasdis.2022.106744 | Included |  |
| Droś J, Segiet N, Początek G, Klimkowicz-Mrowiec A. Five-year stroke prognosis. Influence of post-stroke delirium and post-stroke dementia on mortality and disability (Research Study - Part of the PROPOLIS Study). Neurol Sci. 2024;45: 1109–1119. doi:10.1007/s10072-023-07129-5 | Included |  |
| Narasimhalu K, Ang S, De Silva DA, Wong M-C, Chang H-M, Chia K-S, et al. The prognostic effects of poststroke cognitive impairment no dementia and domain-specific cognitive impairments in nondisabled ischemic stroke patients. Stroke. 2011;42: 883–888. doi:10.1161/STROKEAHA.110.594671 | Included |  |
| Ma Z-Y, Wu Y-Y, Cui H-Y-L, Yao G-Y, Bian H. Factors Influencing Post-Stroke Cognitive Impairment in Patients with Type 2 Diabetes Mellitus. Clin Interv Aging. 2022;17: 653–664. doi:10.2147/CIA.S355242 | Included |  |
| Huang Y, Yang S, Jia J. Factors related to long-term post-stroke cognitive impairment in young adult ischemic stroke. Med Sci Monit. 2015;21: 654–660. doi:10.12659/MSM.892554 | Included |  |
| Lee K, Maeda Y, Shintani Y, Matsuura M, Yamaguchi K, Takayama Y. [Case of post-stroke dementia after left medial occipitoparietal lesion]. Rinsho Shinkeigaku. 2008 Jan;48(1):43-7. Japanese. doi: 10.5692/clinicalneurol.48.43. PMID: 18386631. | Excluded | Different study population |
| Fu Y, Hou X, Feng Z, Feng H, Li L. Research progress in the relationship between gut microbiota metabolite trimethylamine N-oxide and ischemic stroke. Zhong Nan Da Xue Xue Bao Yi Xue Ban. 2024 Mar 28;49(3):447-456. English, Chinese. doi: 10.11817/j.issn.1672-7347.2024.230427. PMID: 38970519; PMCID: PMC11208405. | Excluded | Different study population |
| Rodríguez García PL, Rodríguez García D. Diagnosis of vascular cognitive impairment and its main categories. Neurologia. 2015 May;30(4):223-39. English, Spanish. doi: 10.1016/j.nrl.2011.12.014. Epub 2012 Jun 26. PMID: 22739039. | Excluded | Different study population |
| Ye YS, Yang QT, Zhu DY, Deng KX, Lin HJ, Zhang X, Ji T, Zhuo MZ, Zhang YM. [Effects of moxibustion at Yongquan (KI 1) on cognition function and lower limb motor function in patients with post-stroke cognitive impairment of kidney essence deficiency]. Zhongguo Zhen Jiu. 2023 Sep 12;43(9):1018-22. Chinese. doi: 10.13703/j.0255-2930.20221104-k0001. PMID: 37697876. | Excluded | Different study population |
| Zhan J, Pan R, Guo Y, Zhan L, He M, Wang Q, Chen H. [Acupuncture at Baihui(GV 20) and Shenting(GV 24) combined with basic treatment and regular rehabilitation for post-stroke cognitive impairment:a randomized controlled trial]. Zhongguo Zhen Jiu. 2016 Aug 12;36(8):803-806. Chinese. doi: 10.13703/j.0255-2930.2016.08.007. PMID: 29231563. | Excluded | Different study population |
| Du Y, Zhang L, Liu W, Rao C, Li B, Nan X, Li Z, Jiang H. Effect of acupuncture treatment on post-stroke cognitive impairment: A randomized controlled trial. Medicine (Baltimore). 2020 Dec 18;99(51):e23803. doi: 10.1097/MD.0000000000023803. PMID: 33371155; PMCID: PMC7748352. | Excluded | Different study population |
| Fang R, Duering M, Bode FJ, Stösser S, Meißner JN, Hermann P, Liman TG, Nolte CH, Kerti L, Ikenberg B, Bernkopf K, Glanz W, Janowitz D, Wagner M, Neumann K, Speck O, Düzel E, Gesierich B, Dewenter A, Spottke A, Waegemann K, Görtler M, Wunderlich S, Zerr I, Petzold GC, Endres M, Georgakis MK, Dichgans M; DEMDAS investigators. Risk factors and clinical significance of post-stroke incident ischemic lesions. Alzheimers Dement. 2024 Oct 17. doi: 10.1002/alz.14274. Epub ahead of print. PMID: 39417418. | Excluded | Different study population |
| Rasaholiarison NF, Randrianasolo RO, Rajaonarison LA, Rakotomanana JL, Razafimahefa J, Tehindrazanarivelo AD. Fréquence et caractéristiques des AVC impliquant les artères perforantes dans le Service de Neurologie de l’Hopital Bafelatanana, Antananarivo [Frequency and characteristics of strokes involving the perforating arteries in the Department of Neurology at the Befelatanana General Hospital, Antananarivo]. Pan Afr Med J. 2017 Sep 26;28:76. French. doi: 10.11604/pamj.2017.28.76.13593. PMID: 29255546; PMCID: PMC5724724. | Excluded | Different study population |
| Shimizu H, Nagami S, Takahashi N. [A case of cerebral autosomal dominant arteriopathy with subcortical infarcts and leukoencephalopathy (CADASIL) in which lomerizine hydrochloride was suggested to prevent recurrent stroke]. Rinsho Shinkeigaku. 2014;54(1):22-6. Japanese. doi: 10.5692/clinicalneurol.54.22. PMID: 24429644. | Excluded | Different study population |
| Ihara M. [Post Stroke Dementia]. Brain Nerve. 2016 Jul;68(7):743-51. Japanese. doi: 10.11477/mf.1416200506. PMID: 27395459. | Excluded | Different language |
| Gallucci L, Umarova RM. Kognitive Defizite und Demenz nach Schlaganfall [Post-stroke cognitive deficits and dementia]. Ther Umsch. 2021 Aug;78(6):305-311. German. doi: 10.1024/0040-5930/a001278. PMID: 34291660. | Excluded | Different language |
| Johnen A, Räthe S, Lohmann H, Philipp K, Minnerup J, Wiendl H, Meuth SG, Duning T. Häufigkeit und Risikofaktoren der Post-Stroke-Demenz – eine Beobachtungsstudie zu Schlaganfallpatienten ohne vorbestehende kognitive Defizite [Frequency and risk factors associated with post-stroke dementia-an observational study on stroke patients without premorbid cognitive impairment]. Nervenarzt. 2020 Feb;91(2):131-140. German. doi: 10.1007/s00115-019-00830-x. PMID: 31712835. | Excluded | Different language |
| Xu Q, Lin Y, Geng JL, Li HW, Chen Y, Li YS. [The prevalence and risk factors for cognitive impairment following ischemic stroke]. Zhonghua Nei Ke Za Zhi. 2008 Dec;47(12):981-4. Chinese. PMID: 19134298. | Excluded | Different language |
| Chertcoff AS, Quiroga Narváez J, Saucedo MÁ, Bandeo L, León Cejas L, Uribe Roca C, Fernández Pardal MM, Reisin R, Bonardo P. Impacto psicosocial del ataque cerebral en pacientes jóvenes: una complicación frecuente y habitualmente sub-diagnosticada [Long-term psychosocial impact in young stroke survivors: a frequent but often under diagnosed complication]. Vertex. 2021 Dec;XXXII(154):21-31. Spanish. doi: 10.53680/vertex.v32i154.112. PMID: 35041730. | Excluded | Different language |
| Heupel-Reuter M, Denkinger M, Bauer JM, Voigt-Radloff S. Blutdrucksenkende Behandlung zur Prävention von Schlaganfallrezidiven, schwerwiegenden vaskulären Ereignissen und Demenz bei Patienten mit Schlaganfall oder transitorischer ischämischer Attacke in der Vorgeschichte [Blood pressure-lowering treatment for prevention of recurrent stroke, severe vascular events and dementia in patients with stroke or transient ischemic attack in the past history]. Z Gerontol Geriatr. 2019 Mar;52(2):195-197. German. doi: 10.1007/s00391-019-01521-7. PMID: 30868224. | Excluded | Different language |
| Narita Y, Watanabe Y, Matsumura K, Matsuyama M, Hori K, Kuzuhara S, Ishihara A. [A 59-year-old woman with recurrent convulsive seizures, cerebral infarctions, dementia, and intracranial calcifications]. No To Shinkei. 2000 Jun;52(6):541-52. Japanese. PMID: 10875129. | Excluded | Different language |
| Takahashi K, Yoshizaki K. [Study of the familiar form of vascular dementia (CADASIL)]. Nihon Shinkei Seishin Yakurigaku Zasshi. 2007 Jun;27(3):141-5. Japanese. PMID: 17633526. | Excluded | Different language |
