# Supplementary material for: Effect of post-stroke cognitive impairment and dementia on stroke recurrence and functional outcomes: A systematic review and meta-analysis
Source: PLoS One. 2024 Dec 3;19(12):e0313633. doi: 10.1371/journal.pone.0313633 (PMC11614207; doi:10.1371/journal.pone.0313633)
Supplement: S3 File — (DOCX) [file pone.0313633.s012.docx]

A table of all data extracted from the primary research sources for the systematic review and/or meta-analysis. The table must include the following information for each study:
a. Name of data extractors and date of data extraction
b. Confirmation that the study was eligible to be included in the review.
c. All data extracted from each study for the reported systematic review and/or meta-analysis that would be needed to replicate your analyses.
If data or supporting information were obtained from another source (e.g. correspondence with the author of the original research article), please provide the source of data and dates on which the data/information were obtained by your research group.

| **Recurrence** |
| --- |
| **Univariate** |

| **Study** | **logRR** | **loglci** | **loguci** | **Sample** | **Follow up** | **Country** | **Tool used** | **Cut off category** | **Type PSCI** | **NOS** | **Continent** | **Type** | **Follow cat** | **Sample size** |
| --- | --- | --- | --- | --- | --- | --- | --- | --- | --- | --- | --- | --- | --- | --- |
| Henon 2003 | -0 | -0.03 | 0 | 202 | 3 | France | IQCODE | 104 | Dementia | 8 | Europe | Dementia | 3 | ≥ 250 |
| Li 2020 | 0.2 | -2.81 | 3.2 | 185 | 3 | China | MoCA-CS | 26 | Cognitive impairement | 7 | Asia | Cognitive impairement no dementia | 3 | <250 |
| Nakano 2015 | -0.3 | -3.5 | 2.7 | 66 | 12 | Japan | HDS-R |  | Dementia | 7 | Asia | Dementia | 12 | <250 |
| Sibolt 2012 | 0.7 | 0.38 | 1 | 486 | 12 | Finland | DSM - III |  | Dementia | 8 | Europe | Dementia | 12 | ≥ 250 |
| Kwan 2021 | 0.33 | -0.05 | 0.7 | 1528 | 45 | USA | CASI | 86 | Global Impairment | 9 | America | Cognitive impairement no dementia | 45 | ≥ 250 |
| Yaghi 2020 | -0.1 | -0.12 | 0 | 393 | 6 | USA | MoCA-CS | 26 | Dementia | 7 | America | Dementia | 6 | ≥ 250 |
| Schmidt 2022 | 0.5 | 0.19 | 0.8 | 246 | 60 | USA | 3MS | ≤ 88 | global cognitive impairment | 8 | America | Cognitive impairement no dementia | 60 | <250 |
| Narasimhalu 2011 | 0.71 | 0.19 | 1.2 | 419 | 36 | Singapore | Neuropsychological Test Battery |  | Cognitive impairement no dementia | 7 | Asia | Cognitive impairement no dementia | 36 | ≥ 250 |
| Ma 2022 | 1.96 | 0.73 | 3.4 | 161 |  | China | PSCI |  | Cognitive impairement | 6 | Asia | Cognitive impairement no dementia |  | <250 |
| Huang 2015 | 0.5 | 0.12 | 0.9 | 350 | 60 | China | TICS-m | <31 | Cognitive impairement | 8 | Asia | Cognitive impairement no dementia | 60 | ≥ 250 |

| **Functional outcome** |
| --- |
| **Univariate** |

| **Study** | **loghr** | **loglci** | **loguci** | **Sample size** | **Duration of follow up** | **Country** | **Cut off** | **Cut off category** | **Type** | **NOS** |
| --- | --- | --- | --- | --- | --- | --- | --- | --- | --- | --- |
| Liao 2022 | 0.73 | 0.36 | 1.1 | 1064 | 12 | China | mRS | ≥ 2 | Functional outcome | 8 |
| Liao 2022 | 0.61 | 0.32 | 0.9 | 1064 | 12 | China | SIS-16 | < Q1 | physical and social functioning | 8 |
| Li 2020 | 2.53 | 0.94 | 4.1 | 185 | 3 | China | mRS | ≥ 2 | functional outcome | 7 |
| Dros 2023 | 1.69 | 1.2 | 2.2 | 345 | 60 | Poland | mRS | ≥ 2 | functional outcome | 7 |
| Huang 2015 | 0.67 | 0.37 | 1 | 350 | 60 | China | TICS-m | <31 | Cognitive impairement | 8 |

| **Mortality** |
| --- |
| **Univariate** |

| **Study** | **logRR** | **loglci** | **loguci** | **Sample size** | **Duration of follow up** | **Country** | **Tool used** | **NOS** |
| --- | --- | --- | --- | --- | --- | --- | --- | --- |
| Henon 2003 | 0.6 | 0.1 | 1.1 | 202 | 6 | France | IQCODE | 8 |
| Kwon 2019 | 0.69 | 0.18 | 1.2 | 376 | 48 | Korea | MMSE | 7 |
| Dros 2023 | 1.6 | 1.09 | 2.1 | 345 | 60 | Poland | IQCODE | 7 |
| Narasimhalu 2011 | 1.86 | 0.81 | 2.9 | 419 | 36 | Singapore | Neuropsychological Test Battery | 7 |
| Kwan 2021 | 0.41 | -0.1 | 0.9 | 1528 | 45 | USA | CASI | 9 |
| Schmidt 2022 | 0.12 | -0.38 | 0.6 | 453 | 60 | USA | 3MS | 8 |

| **Recurrence Multivariate** |  |  |  |  |  |  |  |  |  |  |  |
| --- | --- | --- | --- | --- | --- | --- | --- | --- | --- | --- | --- |
| **Study** | **logRR** | **loglci** | **loguci** | **Sample size** | **Duration of follow up** | **Sample size category** | **Country** | **Tool used** | **Cut off category** | **Type** | **NOS** |
| Sibolt 2012 | 0.61 | 0.23 | 0.95 | 486 | 12 |  | Finland | DSM - III |  | Dementia | 8 |
| Yaghi 2020 | -0.06 | -0.1 | 0.01 | 393 | 6 |  | USA | MoCA-CS | 26 | Dementia | 7 |
| Narasimhalu 2011 | 0.51 | -0.07 | 1.09 | 419 | 36 |  | Singapore | Neuropsychological Test Battery |  | Cognitive impairement no dementia | 8 |

| **Functional outcome Multivariate** |  |  |  |  |  |  |  |  |  |  |  |
| --- | --- | --- | --- | --- | --- | --- | --- | --- | --- | --- | --- |
| **Study** | **loghr** | **loglci** | **loguci** | **Sample size** | **Duration of follow up** | **Sample size category** | **Country** | **Cut off** | **Cut off category** | **Treatment** | **NOS** |
| Liao 2022 | 0.32 | -0.12 | 0.77 | 1064 | 12 | 1064 | China | mRS | ≥ 2 | functional outcome | 8 |
| Liao 2022 | 0.34 | 0.02 | 0.67 | 1064 | 12 | 1064 | China | SIS-16 | < Q1 | physical and social functioning | 8 |
| Li 2020 | 1.99 | 0.03 | 3.95 | 185 | 3 | 194 | China | mRS | ≥ 2 | functional outcome | 7 |
| Dros 2023 | 0.69 | 0.09 | 1.29 | 345 | 60 | 345 | Poland | mRS | ≥ 2 | functional outcome | 7 |

| **Mortality** |  |  |  |  |  |  |  |  |
| --- | --- | --- | --- | --- | --- | --- | --- | --- |
| **Multivariate** |  |  |  |  |  |  |  |  |
| **Study** | **logRR** | **loglci** | **loguci** | **Sample size** | **Duration of follow up** | **Sample size category** | **Country** | **NOS** |
| Kwon 2019 | 0.54 | -0.09 | 1.19 | 376 | 48 |  | Korea | 7 |
| Dros 2023 | 0.52 | -0.12 | 1.18 | 345 | 60 |  | Poland | 7 |
| Narasimhalu 2011 | 1.18 | 0.05 | 2.31 | 419 | 36 |  | Singapore | 7 |
| Schmidt 2022 | -0.1 | -0.63 | 0.41 | 453 | 60 |  | USA | 8 |
