# Supplementary material for: Effect of post-stroke cognitive impairment and dementia on stroke recurrence and functional outcomes: A systematic review and meta-analysis
Source: PLoS One. 2024 Dec 3;19(12):e0313633. doi: 10.1371/journal.pone.0313633 (PMC11614207; doi:10.1371/journal.pone.0313633)
Supplement: S4 File — (DOCX) [file pone.0313633.s013.docx]

A table showing the completed risk of bias and quality/certainty assessments for each study or outcome. Please ensure this is provided for each domain or parameter assessed. For example, if you used the Cochrane risk-of-bias tool for randomized trials, provide answers to each of the signalling questions for each study. If you used GRADE to assess certainty of evidence, provide judgements about each of the quality of evidence factor. This should be provided for each outcome.

| **First name, year** | **Consecutive or random sampling of cases** | **non-random sampling** | **Sample size** | **Non-Response rate** | **Ascertainment of the screening/surveillance tool** | **Comparability** | **Assessment of the outcome** | **Statistical test** | **Quality of study (NOS)** |
| --- | --- | --- | --- | --- | --- | --- | --- | --- | --- |
| Henon 2003 | 1 | 1 | 1 | 0 | 1 | 1 | 2 | 1 | 8 |
| Li 2020 | 1 | 1 | 1 | 0 | 1 | 0 | 2 | 1 | 7 |
| Nakano 2015 | 1 | 0 | 1 | 0 | 1 | 1 | 2 | 1 | 7 |
| Sibolt 2012 | 1 | 1 | 1 | 1 | 0 | 1 | 2 | 1 | 8 |
| Kwan 2021 | 1 | 1 | 1 | 0 | 1 | 1 | 2 | 1 | 8 |
| Yaghi 2020 | 1 | 0 | 1 | 0 | 1 | 1 | 2 | 1 | 7 |
| Schmidt 2022 | 1 | 1 | 1 | 1 | 0 | 1 | 2 | 1 | 8 |
| Narasimhalu 2011 | 1 | 0 | 1 | 0 | 1 | 1 | 2 | 1 | 7 |
| Ma 2022 | 1 | 1 | 1 | 1 | 1 | 0 | 0 | 1 | 6 |
| Huang 2015 | 1 | 1 | 1 | 0 | 1 | 1 | 2 | 1 | 8 |
| Liao 2022 | 1 | 1 | 1 | 0 | 1 | 1 | 2 | 1 | 8 |
| Dros 2023 | 1 | 0 | 1 | 0 | 1 | 1 | 2 | 1 | 7 |
| Kwon 2019 | 1 | 0 | 1 | 1 | 1 | 0 | 2 | 0 | 6 |
